# How I do it – sole innominate cannulation for acute type A aortic dissection

**DOI:** 10.1186/1749-8090-7-125

**Published:** 2012-11-20

**Authors:** Pankaj Kaul

**Affiliations:** 1 Consultant Cardiac Surgeon Leeds General Infirmary, Great George Street, Leeds LS1 3EX, UK

## Abstract

We describe sole direct innominate cannulation for arterial return for establishing both cardiopulmonary bypass and selective antegrade cerebral perfusion in the repair of acute type A dissection and compare it with femoral, axillary, direct aortic and apical cannulations. We believe innominate cannulation has all the advantages of right axillary cannulation and none of its disadvantages. It can be used in all patients in whom innominate artery is not dissected, obstructed, calcified or otherwise diseased.

## Background

Acute aortic dissection affects 5.2 people per million per year – twice the rate of ruptured abdominal aortic aneurysm [1]. It has high natural mortality and morbidity [2]. More than 50% with type A dissection die within 48 hours if untreated. There is considerable debate about the ideal sites for cannulation for arterial return for establishment of cardiopulmonary bypass during repair of type A dissection.

The conventional femoral cannulation with retrograde perfusion is known to have an unpredictable but significant risk of atheroembolism, malperfusion and ischemic leg problems in a small number of patients [3]. Right axillary artery cannulation, although increasingly used, requires a separate incision, involves more time, can be difficult if the artery is small and may be associated with low flows and brachial plexus injury. Direct aortic cannulation, whether by conventional technique, Seldinger method or after venous exsanguination followed by aortic transection and true lumen cannulation, carries a risk of aortic rupture and has a higher incidence of stroke and malperfusion [4,5]. Malperfusion requires rapid sequential resuscitation with cold blood antegrade selective cerebral perfusion (ASCP), cold cardioplegic arrest and lower body perfusion, and can be fatal [4]. Besides, direct true lumen aortic cannuation after venous exsanguination requires a short period of whole body circulatory arrest at normothermia [5]. Transapical cannulation has the disadvantages of significant aortic regurgitation with left ventricular distension and high returns from LV vent and hence a poorer myocardial protection, occasional bleeding from left ventricular apex and an inability to clamp ascending aorta during cooling and hence longer bypass times [6,7]. Double-padded clamps which allow perfusion through the cannula but clamp aorta and special perfusion cannulae with side channels with tips in left ventricle to manage left ventricular distension [8] further complicate the technique. Composite cannulation of femoral artery and right axillary artery, while mitigating some of the disadvantages of the individual sites [9], also, unavoidably, ends up with the risks of both.

We describe innominate artery cannulation for both arterial return and selective antegrade cerebral perfusion for repair of acute type A aortic dissection. This has all the advantages of right axillary artery cannulation and none of the disadvantages. Specifically, it provides selective antegrade cerebral perfusion during lower body circulatory arrest, avoids a separate incision, is quick and can be used in an emergency, avoids brachial plexus injuries and is easy to perform because of the larger size of the artery.

We first described the use of innominate artery during aortic root and arch aneurysm repair in 2009 [10] and have used this cannulation technique for all acute dissection repairs either solely or in a composite manner since. In the rare occasions when innominate artery origin from the arch is dissected or diseased, composite cannulation of femoral artery for arterial return during cardiopulmonary bypass and distal innominate artery or right common carotid artery for ASCP may need to be employed.

In this paper, however, we describe the sole innominate cannulation for the repair of acute type A dissection.

### Surgical technique

All patients who do not have calcified, dissected or otherwise diseased innominate arteries are suitable. Both radial arteries are monitored for systemic blood pressure. All central venous catheters are placed in the right internal jugular or right subclavian vein in case left innominate vein needs to be sacrificed, which might need to be done rarely. Cerebral oxymetry is used as necessary. TOE is employed in all patients and additional information obtained with respect to dissection flap, aortic regurgitation, hemopericardium and ventricular decompensation, if any. General anaesthesia is used in all patients as usual. Neck, chest, abdomen and legs are prepared and draped. A Y connector is put on the arterial line, one limb of Y connected to a short tubing, and the other limb of Y connected to a long tubing which comes to the right side of the sternotomy for connection to the innominate artery later. The arterial tubing is filled with prime and deaired. The arterial circuit is now ready for either sole or composite innominate cannulation.

**Figure 1:**
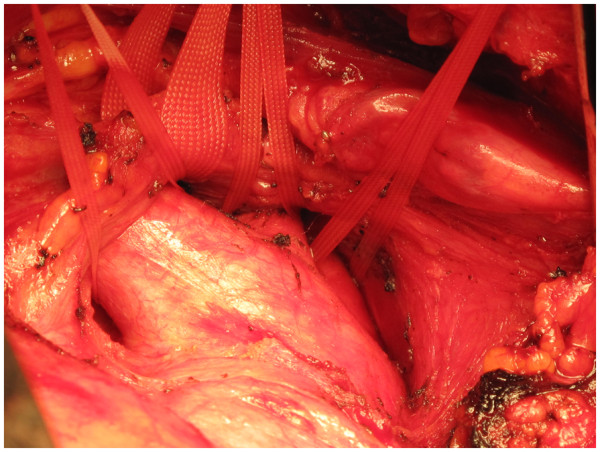
**The left innominate vein (LIV), the innominate artery (IA), the left common carotid artery (LCCA) and the left subclavian artery (LSA) have been dissected and taped in a patient with type A dissection of aorta with a pre**-**existing aneurysm of aortic root and ascending aorta, before pericardium is opened.**

Median sternotomy is made from the sternal notch to the xiphisternum and the incision is extended for 1 inch at the top along the lower end of the left sternomastoid. The avascular plane between two lobes of thymus is developed, thymus completely dissected off the pericardium and all the thymic vein tributaries draining into the left innominate vein divided, thus exposing the entire length of extrapericardial ascending aorta upto arch vessels. The inferior thyroid vein draining into left innominate vein superiorly is divided between clips. The left innominate vein is taped and retracted down or up, exposing the three arch vessels. The innominate artery (IA), the left common carotid artery (LCCA) and the left subclavian artery (LSA) are taped (Figure 1). Occasionally, it is difficult to tape LSA because it dips down posteriorly and to the left in an acute manner, in which case it can be taped on cardiopulmonary bypass. A single or double purse string can be placed on the IA at least one inch away from its origin from the arch. Pericardium is opened and the diagnosis of acute aortic dissection confirmed (2).

**Figure 2:**
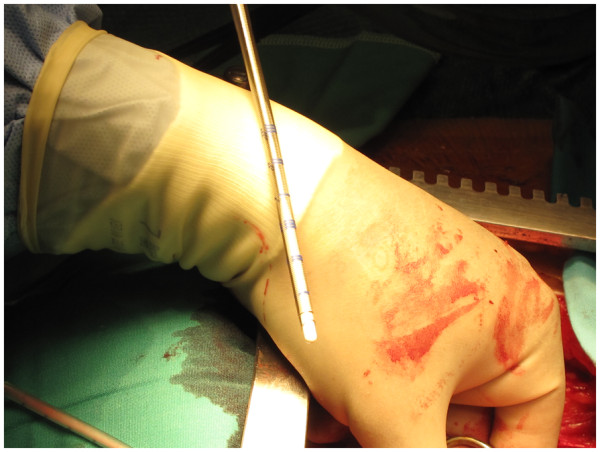
A 20 F wire reinforced Medtronic cannula.

**Figure 3:**
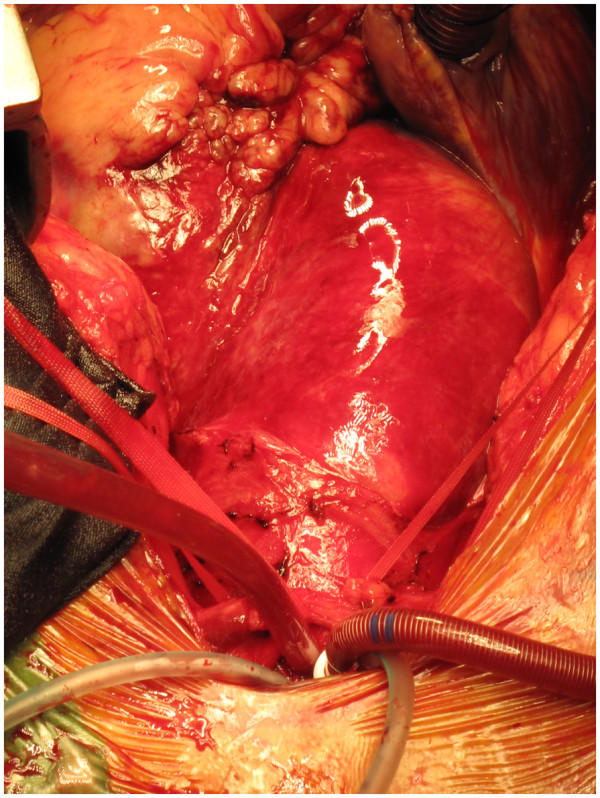
The innominate artery (IA) has been cannulated with 20 F Medtronic cannula which has been advanced into the arch.

**Figure 4:**
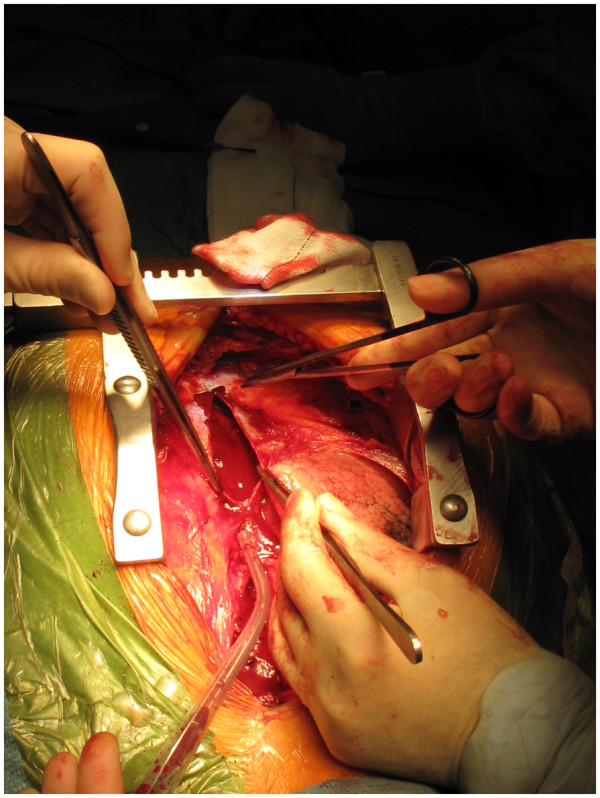
Pericardium is opened in a patient with type A dissection of aorta and as feared the aorta has ruptured with hemopericardium, but innominate artery (IA) is ready for cannulation and establishment of cardiopulmonary bypass.

**Figure 5:**
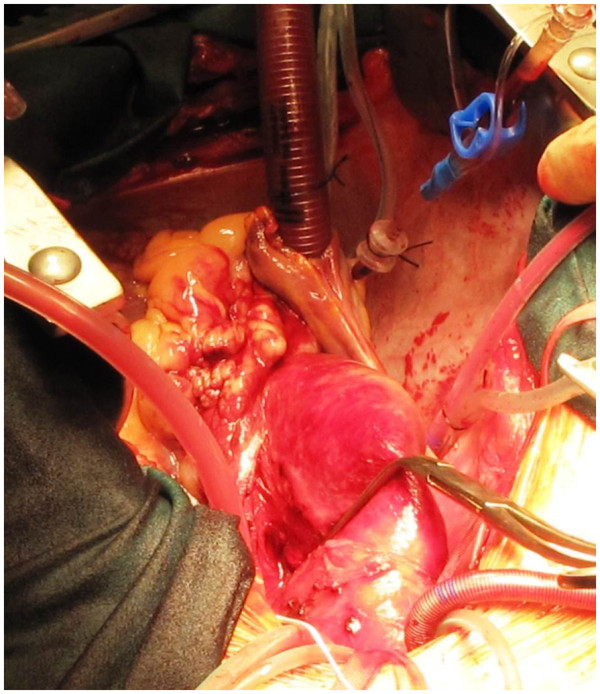
**The dissected aorta is cross clamped midway between the sinotubular junction and innominate artery origin at a place which will be subsequently excised. **This does not pressurise the false lumen owing to antegrade flow in the arch through the innominate cannula.

After total body heparinisation, the IA is cannulated with a 20 F wire reinforced Medtronic cannula (Figure 2). The tip of the cannula is advanced into the arch and the cannula connected to the arterial line from the circuit (Figure 3). The right radial pressure should remain unchanged due to the relatively small diameter of the cannula relative to the IA. The tip of the cannula alternately can be left in the IA facing the arch, but we prefer to insinuate it into the proximal arch. Venous drainage is provided by a two stage cannula introduced into the right atrium. Prior control and placement of purse string in IA helps in instituting expeditious cardiopulmonary bypass if aorta has ruptured (Figure 4), or ruptures after opening the pericardium or if heart decompensates due to hemopericardium, acute aortic regurgitation or coronary dissection. Left ventricle is vented through a catheter placed through right superior pulmonary vein or directly through left ventricle, or sometimes simply through pulmonary artery. Patient is cooled to 17 C. Aorta is clamped well before 17 C, midway between sinotubular junction and the IA in an area which is unquestionably going to be excised (Figure 5). Just before aorta is clamped, preferably when the pump pressures are reduced preparatory to the clamp, it is important to manually confirm that the aortic cannula is truly in the arch and not lying in the ascending aorta where it might be inadvertently clamped. Aorta is incised, the false and true lumens of aorta are entered and heart arrested with cold blood cardioplegia delivered antegradely directly into coronary ostia and retrogradely into coronary sinus. At least 1 litre of cardioplegia is given, but more is given if myocardial temperature in either left or right coronary territories is greater than 15C.

Aortic valve, aortic root, coronary ostia and ascending aorta are inspected and a decision taken regarding whether a supracoronary anastomosis with aortic valve resuspension, a Bentall or modified Bentall operation, or a valve sparing remodelling or reimplantation procedure needs to be done. The requisite proximal procedure is completed. By this time whole body temperature has cooled down to 17C. The flow in the IA cannula is momentarily reduced, the IA cannula is withdrawn into the IA and then redirected so that its tip faces towards the IA bifurcation cranially. The flow in the cannula is re-established at 10 ml/kg/min and the tapes on IA, LCCA and LSA gently snugged down. Head is packed in ice and head end of the table lowered. Aortic cross clamp is removed. The LCCA and the LSA tapes are momentarily loosened to inspect the backflow. If backflow is less than copious, LCCA alone or LCCA and LSA are cannulated with DLP cannulae and the two cannulae infused with arterial blood at the rate of 5ml/kg/min while reducing the IA flow to 5ml/kg/min. We try and maintain a right radial pressure around 50 mm Hg even if that involves increasing the total ASCP flow above 10ml/kg/min. Copious backflow from the LCCA and the LSA is suggestive of a competent circle of Willis, and under such circumstance we do not use additional LCCA/LSA ASCP, unless left radial pressure is below 40 mm Hg. If it is elected to use only IA ASCP, additional confirmation regarding left cerebral perfusion can be obtained by cerebral oxymetry. However, we find bilateral radial arterial pressures and backflow rates from LCCA and LSA quite reliable to determine the need for contralateral ASCP.

**Figure 6:**
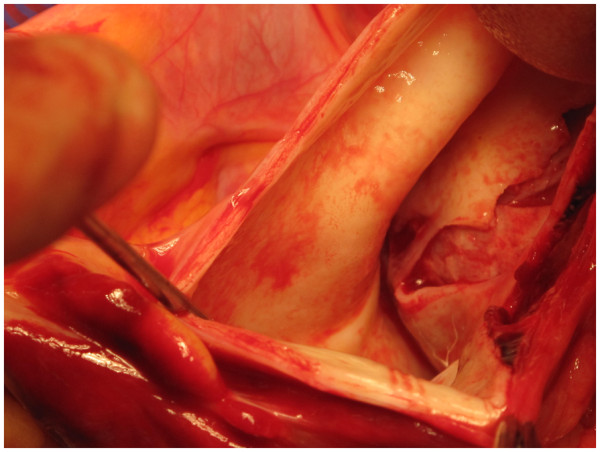
**Long intimal tear in ascending aorta extending to just below the innominate artery. ** Whole of ascending aorta and whole of intimal tear must be excised in repair of type A dissection.

**Figure 7:**
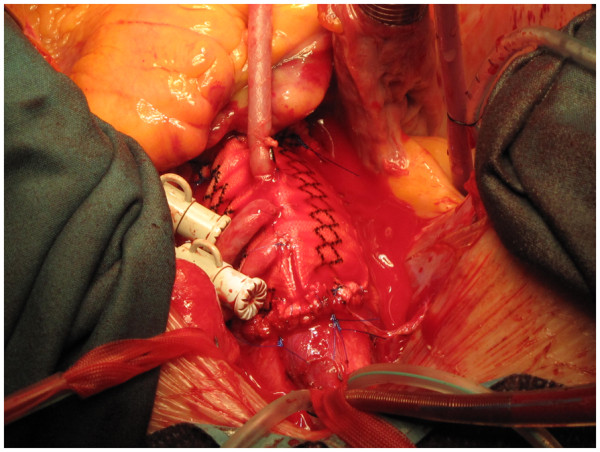
**Retrograde dissection of ascending aorta from descending aortic intimal tear with pre**-**existing coronary artery disease. **Repair included supra-coronary replacement of ascending aorta, suture repair of descending aortic tear and CABG X 3.

The principle for acute dissection surgery continues to be excision of the ascending aorta *and* intimal tear. If the intimal tear is in the convexity of the arch, total arch and ascending aortic replacement are indicated. If intimal tear is in the undersurface of the arch and the origin of the arch vessels is unaffected, ascending aortic and hemiarch replacement may suffice (Figure 6). If, however, the intimal tear is in the descending aorta and there has been a retrograde extension of the dissection into the ascending aorta, then a number of possibilities of complex management arise. If the operator is well versed in arch surgery, one stage in continuity excision of the proximal descending aorta bearing the intimal tear, arch and whole of ascending aorta, with reconstruction of arch vessels is indicated [11]. Repair of the descending intimal tear from within the aorta and replacement of ascending aorta may be possible in selected cases (Figure 7), but is less definitive [12]. Alternately, either replacement of ascending aorta and arch, reconstruction of arch vessels and frozen elephant trunk exclusion of the descending intimal tear would be an option [13]. Chen et al. described a hybrid one stage conventional ascending aortic replacement and open placement of a triple branched self-expandable nitinol stent graft with a polyester vascular graft fabric into proximal descending aorta, arch and the three arch vessels [14]. All our patients, in whom sole innominate cannulation was used have required ascending aortic with or without hemiarch replacement. We have used composite innominate cannulation (with additional femoral arterial cannulation) in patients who had retrograde dissection from a descending aortic intimal tear into ascending aorta [12] and composite right common carotid artery cannulation (with additional femoral arterial cannulation) in a patient with innominate artery dissection and rupture into right pleural cavity [15]. However, the remaining description outlines the procedure we follow when the intimal tear is in ascending aorta or the inferior surface of the arch.

**Figure 8:**
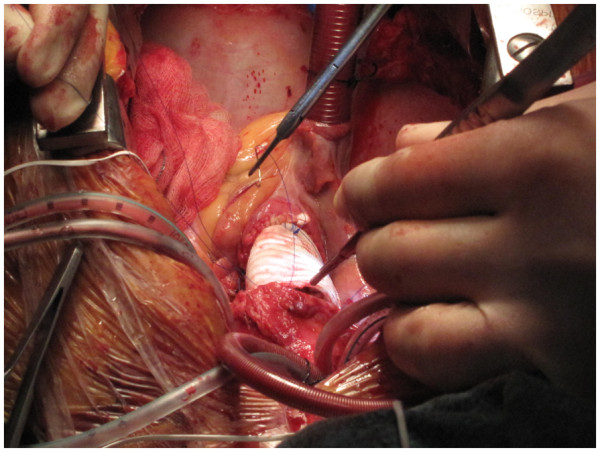
**Open distal anastomosis with lower body circulatory arrest **(**LBCA**) **at 17 C and unihemispherical antegrade selective cerebral perfusion **(**ASCP**) **through the innominate cannula which has been redirected cranially. **Decision for unihemispherical ASCP guided by adequate bilateral radial pressures, cerebral oxymetry and copious backflows from LCCA and LSA.

**Figure 9:**
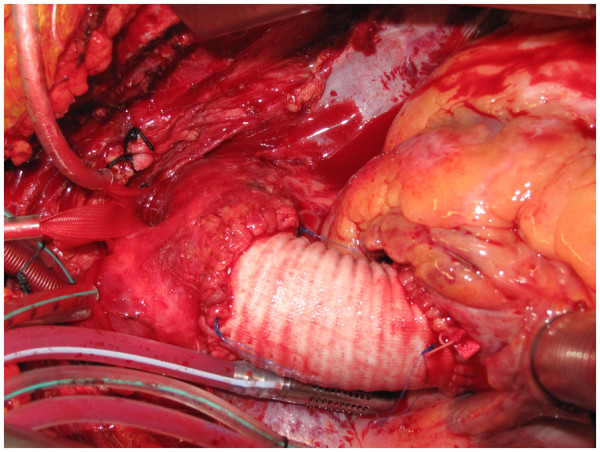
The completed dissection repair, the arch vessel tapes have been unsnugged and the innominate cannula redirected into the arch again.

**Figure 10:**
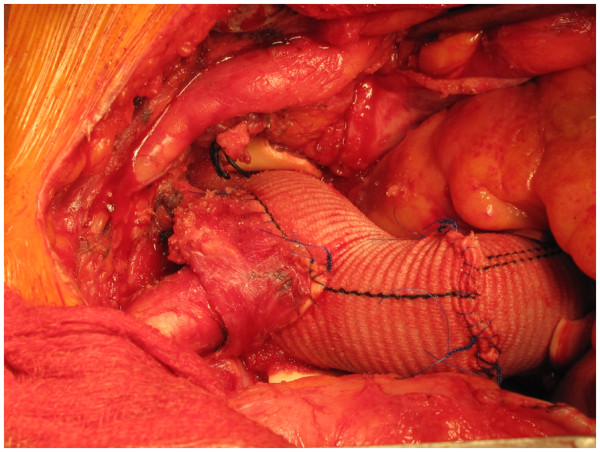
Aortic root, ascending aorta and total arch replacement in a patient with aneurysmal disease using composite innominate cannulation.

The whole of the ascending aorta and the intimal tear are excised. The distal end, of the Dacron graft when aortic valve has been preserved (Figure 8), or the mechanical or biological composite valve graft conduit when aortic valve and root have been replaced, as the case may be, is anastomosed to the remnant of the arch. If separate LCCA and LSA cannulations have been used in addition to IA cannulation for bihemispherical ASCP, flow through LCCA and LSA is stopped, cannulae removed and deairing carried out just before the last few stitches of the distal anastomosis are being placed. The innominate cannula is again withdrawn from the IA and repositioned so the tip again lies in the proximal arch while deairing through the graft is continued and full flows resumed (Figure 9). The customary precaution of checking both radial pressures is taken while repositioning the innominate cannula. If arch is to be replaced, the author will use a separate Dacron graft for the descending aortic anastomosis, reimplant all the arch vessels on a patch and anastomose the two grafts after routine deairing, as he does in patients with extended root, ascending aortic and arch aneurysms (Figure 10). If, however, arch vessels are diseased or involved in dissection, these could be individually implanted on to the ascending aortic graft using an interposition graft.

Patient is slowly rewarmed to 36C. Any additional coronary artery bypass grafting is carried out during rewarming. Bypass is discontinued in the usual manner.

## Discussion

We first described sole innominate cannulation for arterial return and bihemispherical ASCP in a patient with arch and ascending aortic aneurysm with bovine arch variant anatomy in 2009 [10] and have been using sole or composite innominate artery cannulation for repair of acute aortic dissection since [12]. The technique involves introducing a low profile, thin walled cannula through the innominate artery into the proximal arch for instituting cardiopulmonary bypass with antegrade flow in the aorta. The same cannula is redirected cranially to provide antegrade selective cerebral perfusion during lower body deep hypothermic circulatory arrest. Once the repair is complete, the cannula is once again redirected into the arch and whole body circulation resumed. Even when the cannula traverses the proximal IA with its tip in the arch, during total CP bypass, due to the fact that the cannula is less than half of the diameter of the IA, there is sufficient blood going around the cannula from the arch into the innominate artery as evidenced by a normal blood pressure in the right radial cannula and absence of a gradient between right and left radial pressures. Alternately, the cannula may only be redirected cranially or caudally as required without manipulating it in or out of the proximal arch, but we prefer the technique we have described earlier.

Innominate artery cannulation has advantages over all other cannulation techniques.

It provides selective antegrade cerebral perfusion during lower body circulatory arrest as during axillary artery cannulation but avoids a separate incision, can be used quickly in an emergency and avoids brachial plexus injuries. The exposure is easy to perform because of the larger size of the artery and is not made difficult by the size or shape of the patient. It also avoids any vascular complications occasionally associated with a small or thin walled axillary artery.

Compared to femoral artery cannulation, it is quicker to perform, avoids leg incision and avoids the vascular and ischemic complications of leg associated with cannulation and/or long bypass times. It provides antegrade flow at all stages of operation and thus avoids atheroembolism from the abdominal and thoracic aorta in cranial or myocardial circulations, well known with femoral artery cannulation. Innominate cannulation has the additional advantage that aorta can be cross clamped without running the risk of pressurising the false channel further as happens during retrograde flow with femoral arterial cannulation.

IA cannulation avoids the risk of rupture of aorta associated with direct aortic cannulation by conventional or Seldinger techniques. There is no period of circulatory arrest at any temperature with innominate cannulation in contradistinction to a variable period of circulatory arrest at normothermia during direct aortic cannulation after venous exsanguination, aortic transection and true lumen cannulation.

It provides antegrade flow in the arch like the transapical cannulation, but does not affect myocardial preservation owing to aortic regurgitation and ventricular distension encountered almost always with transapical cannulation. Transapical cannulation is associated with higher bypass times as no aortic surgery can be done during the cooling period and so the distal anastomosis cannot start till circulatory arrest has been established and the transapical cannula removed. The distal anastomosis always requires to be done before the proximal anastomosis. Transapical cannulation is also associated with bleeding problems at the apex occasionally.

The innominate cannulation site is always under surgeon’s eye unlike the femoral and the axillary cannulation sites, so that avoidable complications like blood loss and kinking of the cannulae can be detected in time. Also, any extension of dissection into the innominate artery can be visually confirmed and alternate cannulation site chosen, which is why we always employ a Y connector on the arterial line. On the other hand, if right axillary cannulation is used, an innominate dissection might first declare itself as malperfusion at institution of cardiopulmonary bypass [16]. Alternately, innominate artery dissection [17] or dissection and rupture [15] presenting as right hemispherical malperfusion might be salvageable by composite femoral and innominate artery perfusion if distal IA is intact [17] or femoral and right common carotid artery perfusion when distal IA is ruptured [15].

Svensson et al. reported on stroke risk with different cannulation sites for circulatory arrest in 1336 patients, 24 of which had innominate arterial cannulation and found best results with axillary artery with side graft (299 patients) with mortality of 7% and stroke risk of 4% [18]. Eusanio et al. described the use of brachiocephalic trunk for providing antegrade cerebral perfusion during thoracic aortic surgery. The innominate artery was exposed from its origin to bifurcation and an 8 or 10 mm vascular graft anastomosed to it end to side while it was partially side clamped and the arterial line connected to the vascular graft [19]. They extended the same technique to provision of CPB and ACP in 55 patients requiring various forms of aortic surgery, only 1 of whom had acute dissection, and reported 3.6% hospital mortality and 1.8% temporary neurological dysfunction [20]. Augoustides et al. cannulated innominate artery directly in acute type A dissection and severe thoracic trauma [21]. We first described sole innominate cannulation for arterial return and bihemispherical ASCP employing direct cannulation, without using a vascular graft, in a patient with arch and ascending aortic aneurysm with bovine arch variant anatomy in 2009 [10] and have been using sole or composite innominate artery cannulation for repair of acute aortic dissection as well as arch surgery since. Huang described the use of innominate artery with a side graft during arch surgery, thus obviating the need for manipulation during bypass [22]. However, side clamping of the artery at normothermia is required and there is occasional bleeding from the suture line or weeping from the graft. Shi et al. reported 46 cases with repair of type A dissection with ascending aorta and hemiarch replacement combined with stent graft elephant trunk technique using direct innominate cannulation with a 22 or 24 F short-tipped right angled cannula without advancing the cannula into the arch. Operative mortality was 2.2%, there were no strokes and TND grade 2 or above was 6.6% [23].

In conclusion, innominate cannulation can be used in all patients with acute type A dissection for both arterial return and antegrade selective cerebral perfusion unless innominate artery is calcified, dissected or obstructed. The tip of the cannula is insinuated into the arch during CP bypass and cranially towards innominate bifurcation during antegrade selective perfusion. This technique can be used for all open heart surgery when direct aortic cannulation is not feasible and is particularly useful in acute ascending aortic dissections and aortic arch surgery.

## Competing interests

The author declares that he has no competing interests.
